# Preface

**Published:** 1791

**Authors:** 


### PREFACE.

THE prefent Collection of Facts and Obfervations is intended as a fequel to the London Medical Journal. The indulgent manner in which that work was received by the Public, and the numerous and valuable Communications with which the Editor was favoured by his Correfpondents, induced him to perfevere in bringing it out, at ftated quarterly periods, much longer than well fuited his other avocations. But that mode of publication having at length been attended with great inconvenience, both to his profeffional engagements and to the deliberate management of the work, he became deſirous of conducting his future labours in a way more convenient and ſatisfactory to himſelf.

He was aware, that, by making ſuch an alteration in the plan of the Journal as might enable him to continue it at his leiſure, he ſhould retain the advantages which an eſtabliſhed work might be expected to have over a new undertaking; but the reſpect he owed to his readers (many of whom might, perhaps, have conſidered any farther change in the mode of publication as too great a deviation from his original plan) induced him rather to bring the London Medical Journal to a concluſion, and to begin a new Collection, the arrangement of which, ſo far as ſhould relate to the periods of publication, might be better adapted to his other avocations.

The

The London-Medical Journal accordingly ended with the eleventh volume; and the present Collection of MEDICAL FACTS AND OBSERVATIONS is offered to the Public in its ftead. The object of this new work, like that of the Journal, will be to contribute to the improvement and diffufion of medical knowledge; and, like that, it will confift of papers communicated by Correfpondents, and of materials collected from the Tranfactions of learned Societies and other printed works.

This method of blending original obfervations with materials collected from books feems to be the moft proper for a work of this kind, which, while it ferves to excite a fpirit of inquiry, and records interefting facts, is intended to comprife accounts of every important difcovery and improvement that fhall be made in medical fcience.

The

The great Lord Bacon, who complained, with too much reafon, of the few additions made to their art by the medical writers of his time *, recommended Collections of Facts and Obfervations as the beft means of improving the practice of phyfic †; and it is to the method of inveftigating philofophical truth, by induction from accurate experiments, which he fo admirably inculcated, that we are, in a great meafure, indebted for that attention to facts by which medical fcience, in common with every other branch of natural knowledge, hath, fince the days of that truly illuftrious philofopher, been fo much improved.

There is, perhaps, hardly any well-informed perfon, engaged in the practice of phyfic or furgery, to whom opportunities do not now and then occur of adding fome-

* De dignitate et augm. Scient. lib. iv. cap. 2.

† Ibid.

thing thing to our knowledge of diſeaſes; or whoſe mind, from attentive obſervation, may not lead him to ſuggeſt ſome improvement in the modes of treating them: and when it is conſidered that many ingenious men may be willing to communicate the reſult of their experience, in a conciſe and familiar form, who have not leiſure or inclination to compoſe a more elaborate work, the utility of a Collection, like the preſent, which is open to detached facts and obſervations, on any medical ſubject, will, it is preſumed, be ſufficiently obvious.

The Editor flatters himſelf alſo, that by continuing, as in the Journal, to collect a part of his materials from books he ſhall render an acceptable ſervice to the reader. The channels of medical information are now ſo numerous, and in ſo many different languages, that many important obſervations vations probably remain for a long time unknown to persons who are busily employed in the practice of physic, and to whom, of course, they would be the most interesting, but who have not sufficient time or opportunity to consult the several works in which they are to be found.

This remark seems to be more particularly applicable to the Transactions of learned Societies, which, on account of their bulk and price, or the variety of subjects, not immediately connected with physic, of which they treat, are, comparatively speaking, in the hands of few medical readers, although they frequently contain papers with which this class of readers cannot but wish to be acquainted. To collect from such publications, either entire or in an abridged form, the more important observations, relative to the practice of physic and to medical philoſophy, which they contain, ſeems likely, therefore, to be of conſiderable utility; and for the reaſons, juſt now given, the Editor intends alſo to have recourſe, occaſionally, to other printed works, but without profeſſing to give a general review of new medical books. Of theſe, however, a catalogue will be inſerted at the end of each volume.

The Editor propoſes to bring out a part of this Collection as often as he ſhall have got together materials ſufficient to fill about fifteen ſheets in octavo; a volume of this ſize, as it will enable him to make the periods of publication more frequent, ſeeming to be better calculated for the purpoſes of the work than one of greater bulk.

Communications for this work may be addreſſed to Dr. Simmons, Poland Street, London.

